# The effects of the size of nanocrystalline materials on their thermodynamic and mechanical properties

**DOI:** 10.1186/1556-276X-9-516

**Published:** 2014-09-21

**Authors:** Xiaohua Yu, Zhaolin Zhan

**Affiliations:** 1 Faculty of Materials Science and Engineering, Kunming University of Science and Technology, Kunming 650093, People’s Republic of China

**Keywords:** Size effects, Nanocrystalline materials, Thermodynamic, Mechanical, Bond energy

## Abstract

This work has considered the intrinsic influence of bond energy on the macroscopic, thermodynamic, and mechanical properties of crystalline materials. A general criterion is proposed to evaluate the properties of nanocrystalline materials. The interrelation between the thermodynamic and mechanical properties of nanomaterials is presented and the relationship between the variation of these properties and the size of the nanomaterials is explained. The results of our work agree well with thermodynamics, molecular dynamics simulations, and experimental results. This method is of significance in investigating the size effects of nanomaterials and provides a new approach for studying their thermodynamic and mechanical properties.

## Background

Nanocrystalline materials exhibit novel physical and chemical properties which are different from the bulk behavior [1-5]. There are a great many theoretical and experimental investigations showing the size-dependent properties of nanomaterials. The typical trend is that the values of the thermodynamic and mechanical parameters fall with decreasing size of nanoparticles and nanostructure. These parameters include melting entropy and melting point [6-15], Debye temperature [10,16,17], cohesive energy [6,18-23], diffusion activation energy [24,25], amplitude of the thermal vibration [26,27], thermal expansion coefficient [28-30], specific heat [31,32], Young’s modulus [33-36], and mass density [37,38]. All these behaviors are generally explained as a result of the high surface-to-volume ratio of nanomaterials. The proportion of atoms at the surface is no longer negligible and they possess higher energies than atoms in the interior of the particle. Over many decades, a huge volume of data has been established by experiments. However, the mechanism of the size effect is not clear because of the variation of these experimental results. Some excellent models have been developed using classical thermodynamics and modern molecular dynamics. However, most of them focus on only one or two parameters and give different explanations. As a result, there is no common understanding of the mechanism of size effects on nanomaterials. In particular, the question of whether it is possible to correlate the variation of the properties of nanomaterials has rarely received attention.

It is well known that the macroscopic thermodynamic and mechanical properties of crystalline materials are intrinsically determined by the binding energy. Therefore, the change of binding energy is the key to explaining the variation of the thermodynamic and mechanical properties of nanomaterials. In this paper, we present a model based on bond energy. By investigating the energy variation of a nanoparticle, an intrinsic interrelation between the thermodynamic and mechanical properties is achieved, revealing the effects of the size of nanocrystalline materials.

## Theoretical model

**Figure 1:**
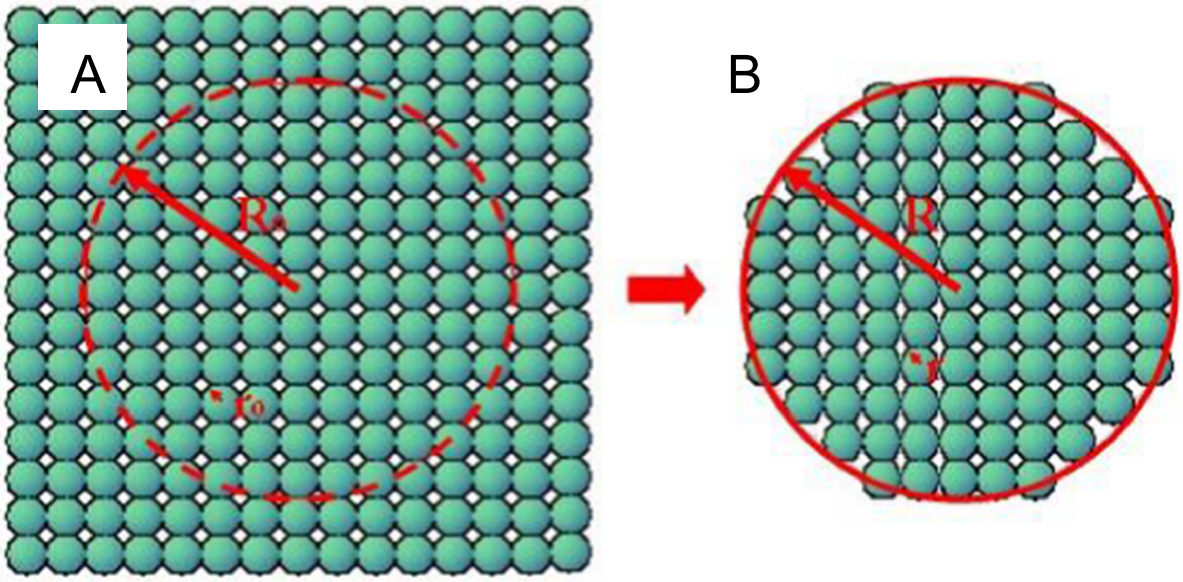
**A perfect crystal (A) and a nanoparticle taken out of it (B). (A)** All the atoms are located at their equilibrium lattice positions. The atomic radius is *r*_*0*_ and the density is *ρ*_*0*_. **(B)** The particle radius is compressed from *R*_0_ to *R*, the average gyration radius reduces to *r*, and the average density increases to *ρ*_*R*_.

Figure 1 is a physical model used for the description of the energy change of a nanoparticle. In a perfect crystal (Figure 1A), there are no defects and all the atoms are located at their equilibrium lattice positions. The atomic radius is *r*_*0*_ and the density of the crystal is *ρ*_*0*_. A nanoscale spherical particle, with radius *R*_*0*_ is taken out of the perfect crystal, as shown in Figure 1B. As bond cleavage of surface atoms takes place, the atoms on the outside of the particle depart from their equilibrium positions, resulting in compression of the particle. The radius of the outside particle decreases to *R.* The average gyration radius is *r* and the average density is *ρ*_*R*_. Since mass is conserved in the above process, the mass of the sphere in the perfect crystal is equal to the mass of the outside particle. Therefore we have

(1) $$\frac{{R_{0}}^{3}}{{r_{0}}^{3}}=\frac{R^{3}}{r^{3}}=\frac{N}{\eta }$$

and

(2) $$\frac{\rho_{0}}{\rho_{R}}={\left(\frac{R}{R_{o}}\right)}^{3}={\left(\frac{r}{r_{o}}\right)}^{3}$$

where *N* is the overall number of atoms in the sphere. *η* is the atomic packing factor and can be determined by calculating the volume of the overall atoms in a unit cell *V*_atoms_, and dividing this by the volume of the unit cell *V*_cell_ as follows, $\eta =\frac{V_{\mathrm{atoms}}}{V_{\mathrm{cell}}}$. The values of *η* are listed in Table 1.

**Table 1:** **The values of the atomic packing factor (*η) *** **for different crystal structures**

**No.**	**Crystal structure**	***η *** **(%)**
1	Body-centered cubic (bcc)	0.68
2	Face-centered cubic (fcc)	0.74
3	Close-packed hexagonal (hcp)	0.74
4	Diamond structure (bct)	0.74

In the above process, the energy of the outside particle would increase by an amount Δ*W*, including the surface energy *W*_1_ induced by the bond cleavage of the surface atoms and the lattice distortion energy *W*_2_ induced by the compression, due to the surface tension of the outside particle. This is summarized by

(3) $$\mathrm{\Delta W}=W_{1}+W_{2}$$

According to thermodynamics, the surface energy of the outside particle is equivalent to the increase of the Gibbs free energy. Therefore, the surface energy *W*_1_ can be calculated from *σ* ⋅ *ΔS.* Δ*S* is the surface area of the outside particle and *σ* is the surface tension. That is

(4) $$W_{1}=\sigma \cdot \mathrm{\Delta S}=\sigma \cdot 4\pi \cdot R^{2}=\sigma \cdot N\cdot 4\pi \cdot {r_{0}}^{2}\cdot \frac{r_{0}}{R}\cdot \frac{\rho_{0}}{\rho_{R}}\cdot \frac{1}{\eta }$$

In addition, the lattice distortion energy *W*_2_ can be calculated by considering the area change of one atom, namely *Nσ*(4*πr*_0_^2^ - 4*πr*^2^), where *N* is the overall number of atoms in the particle. This can be expressed by

(5) $$\begin{array}{ll} W_{2} & =N\cdot \sigma \cdot \left(4\pi \cdot {r_{0}}^{2}-4\pi \cdot r^{2}\right)\\ & =N\cdot \sigma \cdot 4\pi \cdot {r_{0}}^{2}\left[1-{\left(\frac{r}{r_{0}}\right)}^{2}\right] \end{array}$$

Combining Equations 4 and 5, Equation 3 becomes

(6) $$\frac{\mathrm{\Delta W}}{W_{0}}=\frac{r_{0}}{R}\cdot \frac{\rho_{0}}{\rho_{R}}\cdot \frac{1}{\eta }-{\left(\frac{\rho_{0}}{\rho_{R}}\right)}^{\frac{2}{3}}+1$$

where *W*_0_ is defined as *W*_0_ = *N* ⋅ *σ* ⋅ 4 ⋅ *π* ⋅ *r*_0_^2^, referring to the overall bond energy or the standard cohesive energy of the spherical particle in the perfect crystal.

**Figure 2:**
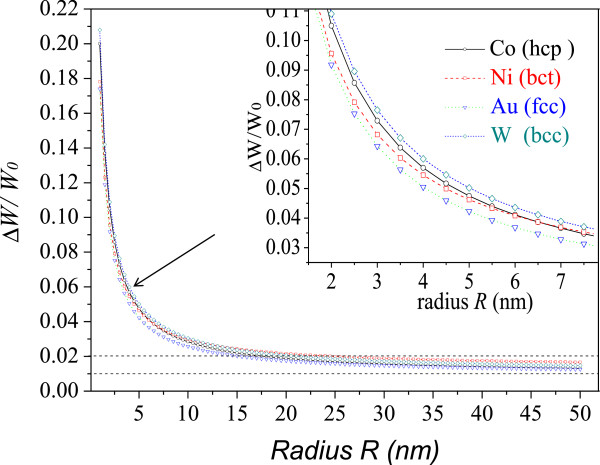
**The change of energy of Co, Ni, Au, and W as a function of the radius of spherical particles.** (*r*_0Co_ = 0.1253 nm, *σ*_Co_ = 1.889 N/m; *r*_0Ni_ = 0.1246 nm, *σ*_Ni_ = 1.823 N/m; *r*_0Au_ = 0.1442 nm, *σ*_Au_ = 1.331 N/m; *r*_0*W*_ = 0.1370 nm, *σ*_*W*_ = 2.534 N/m [39,40]).

We define the term Δ*W/W*_0_ in Equation 6 as the energy variation rate of a nanoscale system. Thus, Equation 6 represents a size-dependent expression of the energy of the nanoparticle. Because Δ*W/W*_0_ is based on the bond energy, which intrinsically influences the macroscopic thermodynamic and mechanical properties of crystal materials, it is reasonable that Δ*W/W*_0_ is used as a general criterion to evaluate the properties of nanomaterials and to predict all those parameters related to bond energy.From Equation 6, it is clear that the energy is in inverse proportion to the radius of the spherical particle. Figure 2 plots the energy of Au, Co, W, and Ni as a function of the radius of the spherical particles. In each case, the rate of change of the energy gradually increases as the radius decreases, for radii greater than 15 nm. When the radius is around 5 to 15 nm, the rate of change of energy apparently increases with the decrease of the radius, being around 2.2% for Co, 2.43% for Ni, 2.03% for Au, and 2.39% for W, respectively, for radii around 15 nm. Sharp increases occur for radii less than 5 nm. The rates of change of energy for Co, Ni, Au, and W are 10.5%, 9.56%, 9.18%, and 10.9%, respectively, for radii around 2 nm.

Based on the principle of conservation of energy, the change of energy of the spherical particle is equal to the increase of the Gibbs free energy and the decrease of the cohesive energy when the spherical particle is removed from the perfect crystal. Therefore Equation 6 can be written as

(7) $$\frac{\mathrm{\Delta W}}{W_{0}}=\frac{\mathrm{\Delta E}}{E_{0}}=\frac{\mathrm{\Delta G}}{G_{0}}=\frac{r_{0}}{R}\cdot \frac{\rho_{0}}{\rho_{R}}\cdot \frac{1}{\eta }-{\left(\frac{\rho_{0}}{\rho_{R}}\right)}^{\frac{2}{3}}+1$$

where Δ*E* and *E*_0_ are the variational and standard cohesive energy of the nanomaterial, respectively. Δ*G* and *G*_0_ are the variational and standard Gibbs free energy, respectively, for a given value of Δ.

By using thermodynamic investigations, molecular dynamics simulations, and experimental methods, considerable research has been carried out in order to investigate the thermodynamic and mechanical properties of nanomaterials. There is much evidence to suggest that some parameters, such as melting point *T*_*m*_, diffusion activation energy *Q*_*d*_, heat of sublimation *L*_*s*_, square Debye temperature *Θ*_*D*_, and Young’s modulus *Y*, can be regarded as being directly proportional to the cohesive energy [1,6,8-30]. Heat capacity 1/*C*_*m*_ is proportional to square Debye temperature *Θ*_*D*_[10]. It is well known that all these properties of crystalline materials are related to the bond energy. Therefore, a general expression can be used to describe the interrelation of the thermodynamic and mechanical parameters of nanomaterials, as follows:

(8) $$\begin{array}{ll} \frac{\mathrm{\Delta W}}{W_{0}} & =\frac{\mathrm{\Delta E}}{E_{0}}=\frac{\mathrm{\Delta G}}{G_{0}}=\frac{\Delta T_{m}}{{T_{m}}_{0}}=\frac{\Delta Q_{d}}{{Q_{d}}_{0}}=\frac{\Delta {C^{-1}}_{m}}{C^{-1}{{}_{m}}_{0}}\\ & =\frac{\Delta L_{s}}{{L_{s}}_{0}}=\frac{\Delta {\Theta_{D}}^{2}}{{\Theta_{D0}}^{2}}=\frac{\mathrm{\Delta Y}}{Y_{0}} \end{array}$$

Combining Equations 7 and 8, this general expression can be simplified to give

(9) $$\frac{\mathrm{\Delta X}\left(R\right)}{X_{0}}=\frac{r_{0}}{R}\cdot \frac{\rho_{0}}{\rho_{R}}\cdot \frac{1}{\eta }-{\left(\frac{\rho_{0}}{\rho_{R}}\right)}^{\frac{2}{3}}+1$$

where, Δ*X* and *X*_0_ are the variational and standard thermodynamic and mechanical parameters, as determined by the bond energy, respectively.

## Discussion

**Figure 3:**
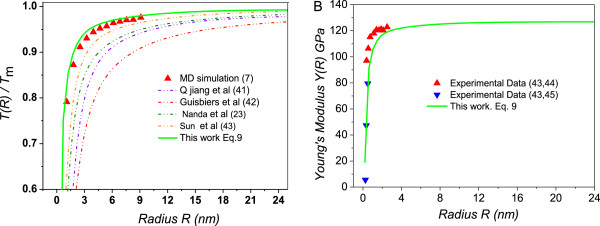
**Size dependence of (A) melting points *T*( *R *)*/T***_***m***_** and (B) Young’s modulus for Cu nanoparticles. (A)** The solid lines denote predictions from our model of *T*(*R*)*/T*_*m*_ in terms of Equation 9, while the black triangles show the molecular dynamics simulation data. Other models [41-43] are also plotted for comparison. **(B)** Agreement between predictions (solid lines) and experimental observations of the size dependence of the Young’s modulus for Cu particles [43-45]. Parameters are given as *r*_0Cu_ = 0.1228 nm, *σ*_Cu_ = 1.534 N/m, *Y*_Cu_ = 128 GPa [39,40].

In Figure 3A, our model (Equation 9) is compared to the result of a molecular dynamics simulation and other models, illustrated using the melting points of copper (Cu) nanoparticles. The best agreement is obtained between our model and the results of molecular dynamics simulation, as the particle radius is increased from 1.08 to 9.10 nm. Other models apparently underestimate the melting points of the copper nanoparticles. The ratio of *T*(*R*)*/T*_*m*_ in our model is slightly higher than the molecular dynamics simulation results, where the size-dependent melting points are lower than those of the experimental data [7]. Figure 3B shows a comparison of the Young’s modulus of copper nanoparticles obtained using Equation 9, with experimental results. Our model predicts the experimental data quite well.

**Figure 4:**
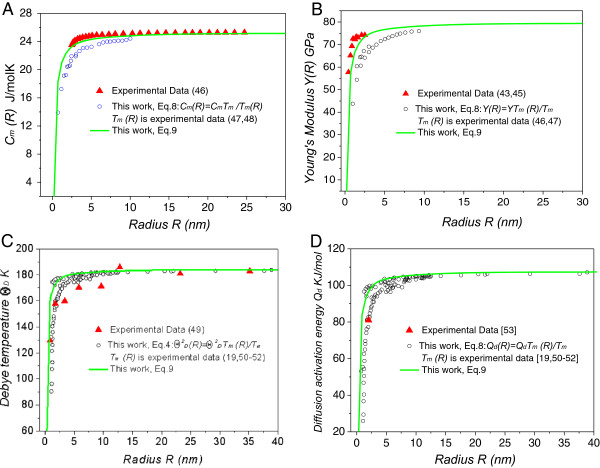
**Effects of size on the mechanical and thermodynamic properties of nanocrystalline materials.** Size dependence of **(A)** heat capacity *C*_*m*_[46-48] and **(B)** Young’s modulus *Y* for Ag [43,45-47], **(C)** Debye temperature *Θ*_*D*_, [49,19-52] and **(D)** diffusion activation energy *Q*_*d*_ for Au [53,19,50-52]. Solid lines refer to parameters calculated from Equation 9 (our model prediction). The white circle is plotted using Equation 4 and experimental data for the melting points *T*_*m*_. The solid triangles show the experimental data (*r*_0Ag_ = 0.1444 nm, *σ*_Ag_ = 1.998 N/m, *C*_*m*Ag_ = 25.36 J/mol · K, *Y*_Ag_ = 80 GPa; *r*_0Au_ = 0.1442 nm, *σ*_Au_ = 1.331 N/m, *Θ*_*D*Au_ = 184.59 K, *Q*_*d*Au_ = 108 KJ/mol [39,40,48,54]).

Equations 8 and 9 reveal the essential relationships between the thermodynamic and mechanical properties of nanomaterials. With Equation 8, we can calculate the thermodynamic and mechanical parameters of nanomaterials using data obtained from either experiments or thermodynamic models. The size dependence of the heat capacity *C*_*m*_ and Young’s modulus *Y* for Ag, the Debye temperature *Θ*_*D*_ and diffusion activation energy *Q*_*d*_ for Au, calculated from Equation 8 using experimental melting point data *T*_*m*_*,* are plotted in Figure 4. The prediction of our model (Equation 9) and the experimental results are also plotted for comparison. Good agreement is obtained between them.

## Conclusions

In summary, we have demonstrated the intrinsic interrelations between the thermodynamic and mechanical properties of nanomaterials using our bond energy model and previous results to characterize aspects of the size effects on nanocrystalline materials. Equation 9 not only presents a new model to better describe the thermodynamic and mechanical properties of nanomaterials, but also provides a new approach to obtain these parameters from others without requiring the formulation and proof of new models. In other words, most of the thermodynamic and mechanical properties of nanomaterials can be predicted by using either experimental data or results of a theoretical analysis. In this way, all factors, such as shape, crystal structure, defects and fabrication processes of nanomaterials, which must be considered when predicting the physical parameters of nanomaterials, can be obtained from experimental data. This is a significant advancement in the investigation and application of nanomaterials.

## Competing interests

The authors declare that they have no competing interests.

## Authors’ contributions

XH proposed the theoretical conception and drafted the manuscript. ZL participated in the theoretical design and helped to draft the manuscript. Both authors read and approved the final manuscript.
